# Short-Time Estimation of Fractionation in Atrial Fibrillation with Coarse-Grained Correlation Dimension for Mapping the Atrial Substrate

**DOI:** 10.3390/e22020232

**Published:** 2020-02-19

**Authors:** Aikaterini Vraka, Fernando Hornero, Vicente Bertomeu-González, Joaquín Osca, Raúl Alcaraz, José J. Rieta

**Affiliations:** 1 BioMIT.org, Electronic Engineering Department, Universitat Politecnica de Valencia, 46022 Valencia, Spain; aivra@upv.es; 2 Cardiac Surgery Department, Hospital Universitari i Politecnic La Fe, 46026 Valencia, Spain; hornero_fer@gva.es; 3 Cardiology Department, Hospital Universitario de San Juan de Alicante, 03550 Alicante, Spain; vbertog@gmail.com; 4 Electrophysiology Section, Hospital Universitari i Politecnic La Fe, 46026 Valencia, Spain; joaquinosca@gmail.com; 5 Research Group in Electronic, Biomedical and Telecommunication Engineering, University of Castilla-La Mancha, 16071 Cuenca, Spain; raul.alcaraz@uclm.es

**Keywords:** atrial fibrillation, catheter ablation, signal processing, chaos theory, coarse-grained correlation dimension, complex fractionated atrial electrograms, nonlinear analysis

## Abstract

Atrial fibrillation (AF) is currently the most common cardiac arrhythmia, with catheter ablation (CA) of the pulmonary veins (PV) being its first line therapy. Ablation of complex fractionated atrial electrograms (CFAEs) outside the PVs has demonstrated improved long-term results, but their identification requires a reliable electrogram (EGM) fractionation estimator. This study proposes a technique aimed to assist CA procedures under real-time settings. The method has been tested on three groups of recordings: Group 1 consisted of 24 highly representative EGMs, eight of each belonging to a different AF Type. Group 2 contained the entire dataset of 119 EGMs, whereas Group 3 contained 20 pseudo-real EGMs of the special Type IV AF. Coarse-grained correlation dimension (CGCD) was computed at epochs of 1 s duration, obtaining a classification accuracy of 100% in Group 1 and 84.0–85.7% in Group 2, using 10-fold cross-validation. The receiver operating characteristics (ROC) analysis for highly fractionated EGMs, showed 100% specificity and sensitivity in Group 1 and 87.5% specificity and 93.6% sensitivity in Group 2. In addition, 100% of the pseudo-real EGMs were correctly identified as Type IV AF. This method can consistently express the fractionation level of AF EGMs and provides better performance than previous works. Its ability to compute fractionation in short-time can agilely detect sudden changes of AF Types and could be used for mapping the atrial substrate, thus assisting CA procedures under real-time settings for atrial substrate modification.

## 1. Introduction

Atrial fibrillation (AF) is the most common cardiac arrhythmia in the developed countries [1], with its rates expected to increase by 2.5 times until year 2050 [2]. Being associated with high mortality risks, the scientific and medical interest of AF is focused both on the understanding of its mechanisms and on candidate treatments [1]. For the latter case, the AF clinical stage, which can be distinguished in paroxysmal, persistent, long-standing persistent, and permanent AF is of high importance. Paroxysmal AF is self-terminating usually within the first 48 hours but can last up to 7 days. Persistent AF (peAF) is characterized by episodes with a duration longer than 7 days, while long-standing peAF lasts at least for a year. Permanent AF exists when its termination is impossible or not recommended and the presence of arrhythmia is accepted by both the clinician and the patient [3].

Common AF treatments include electrical [4] and pharmacological [5] cardioversion and catheter [6] or surgical ablation [7]. After the first reference on the arrhythmogenic role of pulmonary veins (PVs) [8] more than twenty years ago, catheter ablation (CA) targeting PVs foci, a procedure called pulmonary vein isolation (PVI), became the most popular of these treatments. Despite the well-established dominion of this technique up to present, the AF recurrence rate in patients with peAF remains higher than 50% in many cases [9,10].

A possible explanation for the lack of satisfactory results is that foci are triggered in PVs, but re-entrant mechanisms utilize other sites of the atrium and the coronary sinus (CS) musculature for the perpetuation of AF [11]. A vast amount of literature contributes to the effort in understanding these mechanisms [12,13,14]. The most prevalent views attribute the AF either to relatively stable and re-entrant electrical rotors [15] or to epicardial breakthroughs springing from deeper layers of the atrial wall and causing multiple wavefronts due to conduction blocks [16]. The latter perspective is connected to a well-known assumption that peAF is more complicated than paroxysmal due to the structural remodeling of the atria. This remodeling includes, among others, dilatation, scarring, and fibrosis, mostly located on the left side of the atria and remodeled areas are thought to form the pathophysiologic substrate of AF [17].

Fibrosis, in particular, is considered the key change that favors the propagation of irregular and ectopic activations, as it weakens the intercellular connectivity and provokes endo-epicardial electrical dissociation [18,19,20]. Whether fibrosis is the main cause of peAF or the long-term fibrillation provokes it remains unclear [14], whereas it is clear the need for the elimination of the AF substrate. For this purpose, additional ablation based on specific electrogram (EGM) characteristics that possibly indicate the fibrotic tissue, known as substrate modification, is performed. Low-voltage (LV) areas are a candidate target, as reduced signal amplitude is connected to scarring [21]. Relevant studies have shown that additional ablation of LV zones effectively reduces the percentage of AF recurrence [22,23], but the rates of recurrence-free cases depend on the extent up to which LV areas span through the left atrium (LA), which in turn varies among patients [22,23,24].

Highly repetitive re-entrant wavefronts are found in LA sites with high dominant frequency (DF), which have also been investigated as possible remodeled areas [25,26]. However, little or no connection between DF sites in LA and AF elimination was found [27,28]. By contrast, DF in CS has been proven to be able to indicate the patients who need additional ablation [11].

Another one and possibly the most popular indicator of atrial tissue fibrosis is EGM fractionation, which can be considered as a projection of the multiple wavelets scattered through the epicardium and passing simultaneously by the measured point. Highly fractionated EGMs, commonly known as complex fractionated atrial electrograms (CFAEs), are linked to higher incidence rates of re-entrant mechanisms and thus to higher severity of fibrosis [18]. The first successful attempt to distinguish CFAEs that can be targets for substrate modification and effectively eliminate them, reported very high AF recurrence-free rates of 91%, even after one year of follow-up [29].

A high number of studies since then have added precious information in the mapping of AF substrate. Combining CFAEs analysis with LV zones or DF sites has been a subject under investigation as well, with quite controversial results. One study localized CFAEs within CS before and after antral PVI and found DF and EGM complexity to decrease in consistency with ablation efficacy in peAF, implicating the need for simultaneous CS analysis [11]. On the other hand, other studies disprove DF as an independent predictor of ablation guided by highly fragmented EGMs [27,30] or find a spatial proximity of DF sites to areas with CFAEs [30]. Detection of CFAEs in frequency domain highly depends on the technique used. Although Fourier Transform (FT) is the standard method for DF calculation, some alternative techniques have reported improved results. A transform based on the averaging of the various ensembles of a signal has demonstrated reduced measurement error in CFAEs identification [31]. A following study based on this technique [32] showed improved time and frequency resolution in comparison with discrete FT and found the dominant spectral parameters to be higher in peAF than paroxysmal AF, suggesting that AF activation patterns are more regular and stable in peAF patients. Although there are indications that LV zones in sinus rhythm (SR) can adequately predict AF sustaining CFAEs [33], another study has found that there is no necessary correlation between them and LV sites [34].

The comparison between CFAE-based ablation and other AF mapping and ablation methods [35,36] as well as the poor correlation between CFAEs areas and AF drivers [37] has led many studies to consider the ablation of CFAEs to be of little success. These studies, however, do not deny CFAEs as ablation targets. They simply highlight the need for a stricter definition to avoid unnecessary lesions. CFAEs are defined either as EGMs composed by two or more deflections and/or perturbation of the baseline with continuous deflection or as EGMs with a very short cycle length (CL) (≤120 ms) [29]. Correlation between these two definitions is poor, whereas CFAEs locations vary upon the patient and the definition used [30,38].

The demanding need for an accurate and reliable definition of CFAEs is therefore clear, so that their contribution to the AF substrate is loyally depicted. One quite straightforward indicator is the estimation of fractionation level of the EGMs [39]. As it was previously mentioned, low-fractionated EGMs may reflect passive phenomena, but as the fractionation level augments, it is more possible to depict regions of fibrotic tissue. As fractionated EGMs express the superposition of more than one wavelets simultaneously propagating through different directions in the atria, a nonlinear metric may be able to better quantify their fractionation level, and therefore the number and the organization level of the activations that they depict.

This behavior of EGMs as nonlinear dynamical systems has been investigated by several studies [40,41,42,43,44], using a chaos-theory based technique known as correlation dimension (CorDim). CorDim quantifies the level of randomness of a strange attractor and has been used to assess the organization and complexity of EGMs. Hoekstra et al. [40] used coarse-grained CorDim (CGCD) and correlation entropy to classify unipolar epicardial EGMs recorded at the free wall of right atrium by AF Types, as described by Wells et al. [45]. Although this work came out with some very interesting results, the use of unipolar instead of bipolar EGMs narrows down the robustness of the findings. Censi et al. used CGCD and surrogate data in order to assess the nonlinear coupling of cardiac time-series and assumed that the indices used for this purpose may serve for the classification of EGMs by AF Types [41]. In 2014, Luca et al. used CorDim for the quantification of the influence of anti-tachyarrhythmia pacing in model-based AF and suggested it as a discrimination metric of atrial activity organization [43]. Later in 2016, another relevant study used CGCD in right atrial (RA) EGMs before and during catheter ablation for the assessment of peAF complexity [44]. Results revealed a predictive value of this index for the ablation outcome.

Taking advantage of the theoretical and experimental background of all these works, we recruited CGCD as a fractionation index of the atrial EGMs. This work is based on the hypothesis that CGCD is linked to the fragmentation level of EGMs in a pro rata basis and its use can effectively estimate the AF type of patients even in recordings as short as one second in length. The aim of this study is to present a method with real-time implementation capabilities on the mapping devices that can precisely detect areas of high fractionation EGMs as well as short-duration phenomena, so that the AF substrate can be efficiently removed.

The manuscript is organized as follows. Section 2 presents the study population and data acquisition and introduces the theoretical background of the concepts used in the study. It also provides information on the computational parameters and the methods used for the CGCD estimation as well as the statistical analysis, the outcomes of which are presented in Section 3. Next, Section 4 discusses about the main findings and some important issues to be taken into account regarding the study, whereas Section 5 explains the main limitations of the study as well as the context within which some choices have been made. Finally, Section 6 highlights the most relevant aspects of the study introducing the concluding remarks.

## 2. Methods

### 2.1. Study Population and Data Acquisition

**Figure 1:**
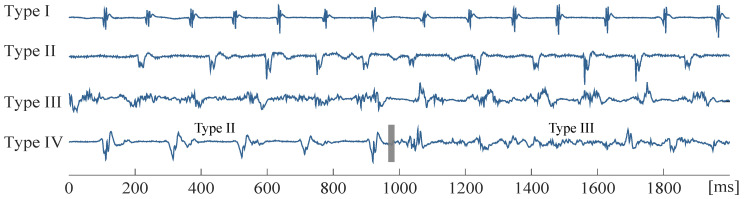
Example of bipolar atrial fibrillation (AF) electrograms (EGMs) of different Types. AF Type IV consists of alternating Type I/II and Type III segments.

The database employed in this study consisted of 119, 10 s duration bipolar EGMs of 22 peAF patients undergoing CA for the first time, after their signed informed consent. Data were obtained using a Labsystem™PRO EP recording system (Boston Scientific, Marlborough, MA, USA) with a sampling frequency set at 1 kHz and a bandpass filter at 0.5–500 Hz. EGMs were visually inspected and classified according to their AF type following Wells’ criteria [45]. There are three main AF Types: AF Type I is characterized by discrete activations with a stable isoelectric line, whereas AF Type II is also characterized by discrete activations, with the baseline presenting perturbations of varying degrees. AF Type III lacks either discrete complexes or isoelectric intervals. There is also AF Type IV, which is consistent with AF Type III, with altering parts of AF Types I or II. Figure 1 shows an example of different AF Types.

In total, 11 EGMs were classified by two expert physicians as Type I, 36 as Type II, and 72 as Type III. Visual classification by AF type can be confusing in cases that an EGM does not clearly belong to an AF type. To make a fair assessment of CGCD as a fractionation index, eight EGMs from each category were selected as the most representative of their type. Additionally, 20 pseudo-real EGMs were created by the concatenation of parts of real EGMs to create some Type IV electrograms.

Data analysis has been performed on three groups. Group 1 consisted of the 24 most representative EGMs, eight of each type, selected by the two experts, as the EGMs that undoubtedly belonged to the assigned type. These EGMs represented the common choice of the experts, with each one of them being blinded to the selection of the other one. Group 2 consisted of all the electrograms of the database, whereas Group 3 contained the 20 pseudo-real Type IV electrograms.

### 2.2. Coarse-Grained Correlation Dimension

Randomness of the dynamics characterizing AF can be assessed by CorDim, a well-known measure of the organization of nonlinear dynamical systems [46]. The main idea of this method claims that by reconstructing the observed time-series in phase space, one can assess its stochastic nature by calculating the distance between elements of the time-series and comparing it with a resolution distance *r*. In other words, a set of nondeterministic (chaotic) points will occupy more space than a set of deterministic ones. The dimensionality of this space is expressed as CorDim.

The first step for the calculation of CorDim is the phase-space reconstruction of the given time-series, with the reconstructed system preserving the dynamical characteristics of the original dataset [47]. More specifically, given a *N*-points long time series $X=(x_{1},x_{2},\ldots ,x_{N})$, one can reconstruct it to the *m* dimensional phase-space using a time delay τ between vectors [40,48]. Phase-space reconstruction of the *p*-th element of *X* will then be

(1) $$Y_{p}^{\left(m\right)}=\left(x_{p},x_{p+\tau },x_{p+2\tau },\ldots ,x_{p+(m-1)\tau }\right),$$

where m=1,2,3… is the embedded dimension and p=1,2,…,N−(m−1)τ.

The second step after phase-space reconstruction is completed is the estimation of the correlation integral [46], which calculates the proportion of pairs of vectors that are closer to each other than a distance *r*,

(2) $$C^{\left(m\right)}\left(r\right)=\frac{2}{N_{ref}\left(N_{ref}-1\right)}\sum_{i=1}^{N_{ref}}\sum_{j>i}^{N_{ref}}\Theta \left(r-∥Y_{i}^{\left(m\right)}-Y_{j}^{\left(m\right)}∥\right),$$

where Θ is the Heaviside function, and ∥·∥ is the Euclidean distance of each pair chosen and $N_{ref}$ is the number of reference points, as a chosen number of the N−(m−1) vectors of Equation (1).

For the computation of CorDim, we search for saturation areas (linear regions) on the double logarithmic plot of $C^{\left(m\right)}\left(r\right)$ as a function of *r*, plotted in sequential embedded dimensions from m=1,2,…,20 [44]. By taking a look at the correlation integral, one can see that CorDim is inversely proportional to the organization of the underlying dynamics. This can be explained by the fact that two strongly associated points will not be very far away from each other, when the reconstructed phase-space faithfully represents the original data. On the other hand, highly irrelevant or weakly associated points will be found in random positions that, when averaged through the whole dataset, will be significantly far away from each other.

In cardiac signals, specially in significantly fragmented EGMs dominated by highly disorganized dynamics, the lack of regions of saturation and, as a result, the incapacity of a reliable description of the reconstructed dynamics by the correlation integral, is always a possibility to be considered [40,44]. A slight variation of the correlation integral, known as CGCD can nevertheless still be used to measure the organization of the dynamics of invasive cardiac recordings [40]. More of a comparative measure between the complexity of the signals than a precise dimension estimator [44], CGCD makes a rough estimation of this complexity at a fixed embedded dimension *m* and a finite resolution distance $r_{cg}$, thus

(3) $$CGCD^{\left(m\right)}\left(r_{cg}\right)=\frac{dln[C^{\left(m\right)}\left(r_{cg}\right)]}{dln(r_{cg})},$$

being the selected nonlinear index applied in the present work to atrial EGMs of AF with the aim to estimate their fractionation. For the calculation of the $CGCD^{\left(m\right)}\left(r_{cg}\right)$ from the double logarithmic plot of $C^{\left(m\right)}\left(r\right)$ as a function of the distance *r*, we need to calculate the derivative of the correlation integral curve, when $r=r_{cg}$. This can be approached by the local slope of the tangent line, passing from the point $(ln\left(r_{cg}\right),ln\left(C^{\left(m\right)}\left(r_{cg}\right)\right)$, calculated through two points, $(ln\left(r_{1}\right),ln\left(C^{\left(m\right)}\left(r_{1}\right)\right)$ and $(ln\left(r_{2}\right),ln\left(C^{\left(m\right)}\left(r_{2}\right)\right)$, surrounding the point $(ln\left(r_{cg}\right),ln\left(C^{\left(m\right)}\left(r_{cg}\right)\right)$ [40].

### 2.3. Selection of Computational Parameters

As mentioned in Section 2.2, for the computation of CGCD, it is necessary to set the following parameters; the embedded dimension *m*, the time lag τ, the distance $r_{cg}$, and the number of reference points $N_{ref}$. The choice of these parameters is very important for both the optimal operation and the discriminative power of CGCD between AF fractionation levels and is hereby discussed in detail.

#### 2.3.1. Embedded Dimension

Correct embedded dimension *m* is important for the loyal resemblance of the reconstructed signal. According to Hoekstra et al. [40], *m* needs to be sufficiently large so that dynamics are faithfully described by the reconstructed signal. In case that *m* is smaller than the dimension of the dynamics under analysis, double logarithmic plot will present no linear regions [48]. Optimal *m* can vary from signal to signal, it is necessary though to set a global *m* value and analyze the dynamics over it. The choice of *m* in previous works was empirical and chosen to m=10 [40,44].

#### 2.3.2. Time Lag

The other factor influencing both resemblance precision of the reconstructed signal and discrimination power through AF fractionation levels is time lag τ. When choosing a small time lag, all points in Equation (1) will tend to become indistinguishable [49]. A common choice for τ is to be equal to the first minimum of the mutual information [40].

#### 2.3.3. Distance in Phase Space

Selection of distance $r_{cg}$ affects both accuracy and precision of the method. By scaling it down, a better control of systematic errors and thus an increase in accuracy is achieved. At the same time, however, statistical errors are augmented and consequently precision drops down. It is important, therefore, to choose $r_{cg}$ as a trade-off between these two parameters. Theiler [50] suggested that systematic and statistical errors are treated as an entity and tried to find the optimal $r_{cg}$ for its reduction. A distance $r_{cg}$ equal to half of the standard deviation of the time-series, normalized by its peak-to-peak amplitude is commonly used [40].

#### 2.3.4. Reference Points

Wise choice of reference points $N_{ref}$ is important for the statistical precision of the dimension analysis. A choice of a small $N_{ref}$ would lead to poor statistical validity [50] and the risk of calculating a correlation integral of zero value would be present for dynamics of high-dimensional chaos. Regarding the lowest limit, $N_{ref}$ equal to 1/3 of total points of the time-series was found to be acceptable [50]. Speaking of the upper limit, the choice must be made taking into consideration the execution time of the algorithm, which significantly increases when the time series is quite long and the percentage of precision improvement that is succeeded.

### 2.4. Data Preprocessing and Analysis

To minimize the influence of signal amplitude on CGCD, each signal was firstly normalized by its root mean square (RMS) value. RMS value is the square of the function that defines the time-series. As signal amplitude varies from recording to recording, normalizing by a standard value would be of no meaning. Using RMS value hence, the time-series is normalized while the information is kept intact.

After that, signal preprocessing continued by using a 3rd-order Butterworth lowpass filter with cut-off frequency at 300 Hz and a wavelet-based denoising technique which reduces effectively high frequency noise [51]. Finally, EGMs were segmented to 1 s intervals. Segmentation of signals in short-time intervals is a choice that will be later discussed.

**Figure 2:**
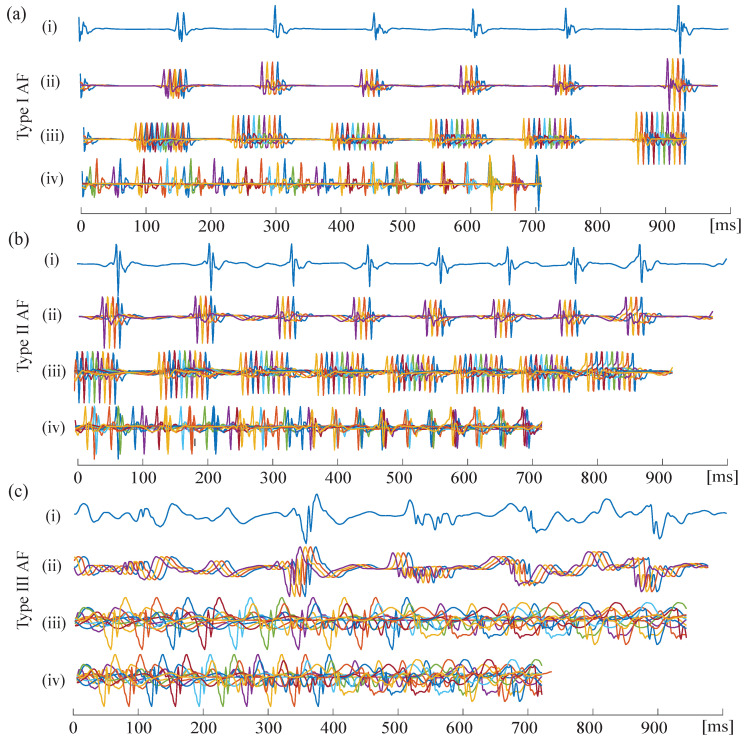
Example of one second segment of (**i**) original and (**ii**–**iv**) reconstructed AF electrograms via CGCD. (**ii**) Reconstructed signal with time lag τ=8 ms, embedded dimension m=4. (**iii**) Reconstructed signal with time lag τ=8 ms, embedded dimension m=10. (**iv**) Reconstructed signal with time lag τ=35 ms, embedded dimension m=10. (**a**) AF Type I, (**b**) AF Type II, and (**c**) AF Type III. Length *p* of reconstructed signal decreases as τ and *m* increase, as can be seen from Equation (1).

CGCD was computed for each time-series at the segmented 1 s intervals and then the final CGCD value was obtained by the median index of all the intervals. Parameter selection of CGCD was made so that the comparative analysis of fractionation of each EGM is optimal. First, the time when mutual information dropped to its first minimum was calculated. Analysis indicated that for our dataset, this value was for τ=8 ms. $N_{ref}$ was firstly set equal to 1/3 of signal length, that is, in our case, 334 points. After multiple trials choosing the 334 embedded vectors randomly from the set of the reconstructed vectors, we concluded to the use of the first 334 vectors of each segment, as this choice provided similar results to the random choice case and a highly improved execution time. Distance $r_{cg}$ was computed for each signal equal to half of its standard deviation, normalized by its peak-to-peak amplitude [44]. Finally, CGCD was computed for different embedded dimensions from m=1,2,…,20 and the dimension providing the most discriminative power and avoiding infinite CGCD values was selected. As an illustration of the process, Figure 2 shows the reconstructed signals of different AF Types for various parameters. Selection of embedded dimension *m* is a procedure that needs extreme care and its choice will be further discussed in Section 4. In this study, dimension m=4 was the optimal choice.

With the aim to specify the optimal parameters, the method was tested using different number of reference points $N_{ref}$. However, these trials did not improve the classification accuracy, while they increased significantly the execution time. The number of reference points was therefore kept to $N_{ref}=334$.

### 2.5. Surrogate Data Analysis

Before applying nonlinear techniques for data analysis, one should look for any indicators of nonlinearity in the dataset. This can be achieved by the method of the surrogate data, where for each time series, a specific number of surrogate signals is created so that they share given linear properties with the original one. Then, one or more nonlinear indices are computed for both the original and the reconstructed series and the statistical differences between them are investigated. Nonlinearity is present in the original dataset if the index differs significantly from the index of the surrogates in most of the time series [52]. Surrogate data analysis is hardly used for the rejection of the existence of chaotic behavior in the time series. On the contrary, the presence of nonlinearity revealed by the surrogate analysis is a strong indicator of chaotic behavior [53].

For each signal, 40 surrogates were created using the iterative amplitude adjusted Fourier transform (iaaFT) [54], corresponding to 95% confidence level. The iaaFT is an alternative of the amplitude adjusted Fourier transform (AAFT) technique [52] with corrected deviations in spectrum and distribution [54] in line with the original data. First of all, the amplitude of the original time series is rescaled in order to have a Gaussian distribution. Afterwards the phases of the reconstructed signals are randomized in a way that conserves the normality of the distribution on average and then the reconstructed signals are rescaled to fit with the amplitude distribution of the original signal. The iaaFT method finishes with the iterative correction described earlier in this paragraph. The produced surrogate signals have the same amplitude distribution and power spectrum with the original signal. The CGCD of the original signals was compared with the CGCD values of the surrogates using a rank-order test. In case of statistically different CGCD values, the null hypothesis of linearity is rejected and the original data are considered nonlinear.

### 2.6. Statistical Analysis

For Groups 1 and 2, CGCD values were used to classify EGMs by AF type. Firstly, one-vs-all receiver operating characteristics (ROC) analysis was used to assess the discrimination by CGCD. Afterwards, a decision tree was used in order to evaluate statistically the discriminative power of CGCD. For this purpose, Matlab^®^ Classification Learner (MathWorks, Natick, MA, USA) performed a coarse-tree analysis with a maximum split of 2, using 10-fold cross-validation. Normality and homoscedasticity of the median values for the three AF Types were tested with Shapiro–Wilk [55] and Levene tests [56], respectively. According to the results of the above tests, statistical differences between the median values of the three AF Types of each group were verified with the Kruskal–Wallis test [57], whereas statistical differences between the median values in pairs of AF Types were also tested, using a Mann–Whitney U test [58] with Bonferroni correction.

**Figure 3:**
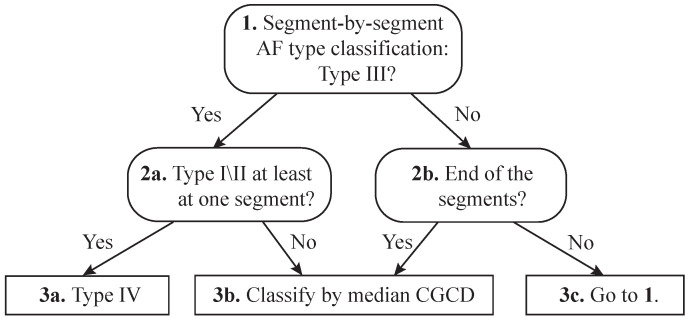
Illustration of algorithm steps and decisions taken for AF Type IV detection on the pseudo-real recordings of Group 3 in the database.

For Group 3 of pseudo-real EGMs, an algorithm assigning the EGMs under analysis to one of the AF Types (I, II, III, and IV) was developed. The algorithm was firstly performing CGCD analysis as described in Section 2.4. Then, using the thresholds obtained by the decision tree analysis on Group 2, CGCD value was assessed at each 1-s segment. If an EGM was classified as Type III by at least one of its segments, segment-by-segment classification by AF type started over on this specific EGM. If at least one of its segments is classified as Type I or II, the EGM is finally assigned to AF Type IV. Figure 3 shows the steps followed for the Group 3 analysis. Evaluation on this group was based on the percentage of pseudo-real EGMs correctly classified as Type IV.

## 3. Results

### 3.1. Surrogate Data Analysis

**Figure 4:**
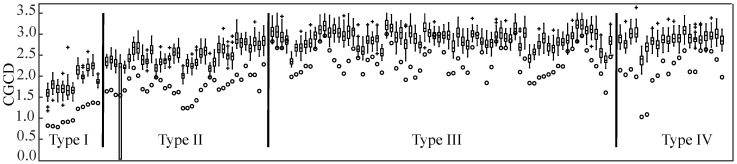
Surrogate data analysis indicating coarse-grained CorDim (CGCD) values for the entire database. Values of original data are presented with a small circle, whereas surrogate values are depicted as boxplots, generated from the 40 surrogates corresponding to each time series.

CGCD values of most of the time series differed significantly from their surrogates. Thus, the application of nonlinear techniques is justified from the presence of nonlinear dynamics, confirmed by the surrogate data analysis. Figure 4 shows the CGCD values for all the AF Types for original and surrogate data, where the index values of the surrogate data are higher than the index values of the original ones.

### 3.2. Statistical Analysis

**Figure 5:**
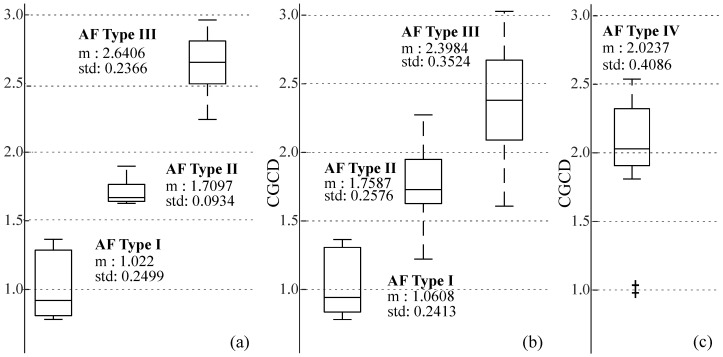
Box plots illustrating the distribution CGCD values as a function of the AF Types, where (**a**) is for the most representative EGMs in Group 1, (**b**) for the whole database in Group 2, and (**c**) for Type IV pseudo-real EGMs in Group 3.

**Figure 6:**
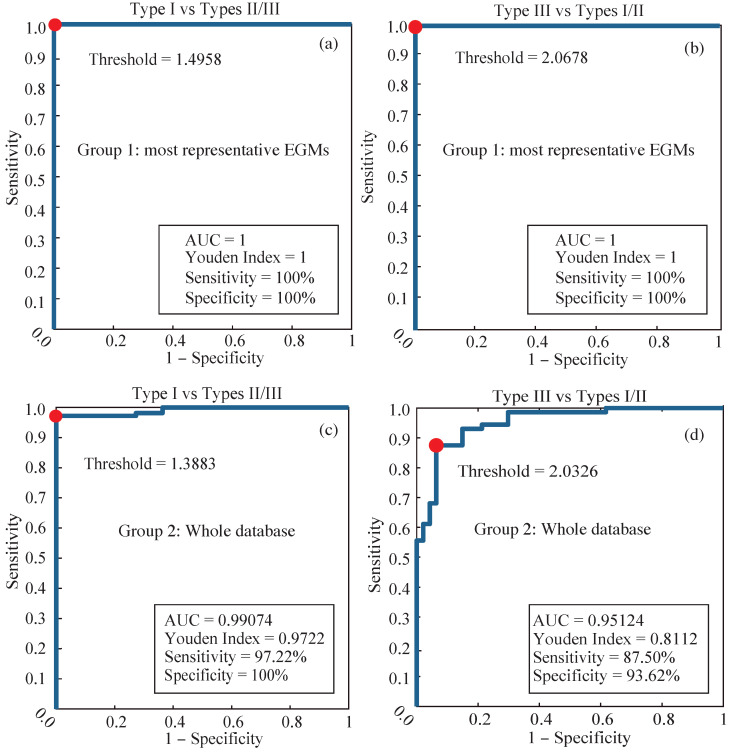
Receiver operating characteristics (ROC) curve analysis of discrimination between AF Types by using CGCD as a fractionation index. (**a**,**b**) Curves for the 24 most representative EGMs in Group 1 and (**c**,**d**) curves for the whole dataset analyzed in Group 2. AUC: area under the ROC curve.

The box plots and ROC curves from the three defined Groups can be seen in Figure 5 and Figure 6, respectively. CGCD showed a very strong discriminative power for the 24 most representative EGMs in Group 1, where all EGMs were classified by AF type in consistency with the visual classification. The area under the ROC curve (AUC) and the Youden index in this group were 1, when the discriminative power of CGCD as a fractionation index between AF Type I and AF Types II and III was tested. The same values were obtained when efficient discrimination between AF Type III and AF Types I and II was tested. Applying ROC curve for discrimination of AF Type II from AF Types I and III was of no meaning, as AF Type II takes values that lie in between AF Types I and III.

For the whole database in Group 2, Figure 5b shows that AF Type II takes some values that may overlap with values of AF Types I and III, as expected. Still, mean values are well discriminated at each AF type. Figure 6c,d show a quite high discriminative power, although the AUC was a little bit lower than Group 1. AF Type I in this group was well discriminated from the other two AF Types, while AF Type III was slightly less well defined, due to the ambiguity of this AF type in some cases. Yet, the AUC was 0.95 and the Youden Index 0.81, which are still quite high.

Finally, as Group 3 contains AF Type IV EGMs, their mean values are mainly in between AF Types II and III, as Figure 5c shows. This suggests that in a CGCD analysis without 1 s segmentation, these EGMs would be classified either as AF Type II or as AF Type III. In the end, AF Types I, II and III of Groups 1 and 2 can be well discriminated according to their box plots shown in Figure 5, whereas AF Type IV CGCD values coincide with the corresponding AF Types II and III.

Statistical differences between AF Types for Groups 1 and 2 as well as between pairs of AF Types, are shown in Table 1. Median CGCD values were statistically different for the AF Types of both groups, both when they were tested separately and in pairs.

In Group 2, 104 (87.39%) EGMs were correctly classified by their AF type, according to the ROC analysis thresholds. Of the 15 wrongly classified EGMs, six belonged to AF Type II (83.33% classified correctly) and nine belonged to AF Type III (87.5% classified correctly). Regarding Group 3, 20 out of 20 pseudo-real EGMs (100%) were correctly classified by AF Type IV, according to the segment-by-segment analysis presented in Figure 3.

**Figure 7:**
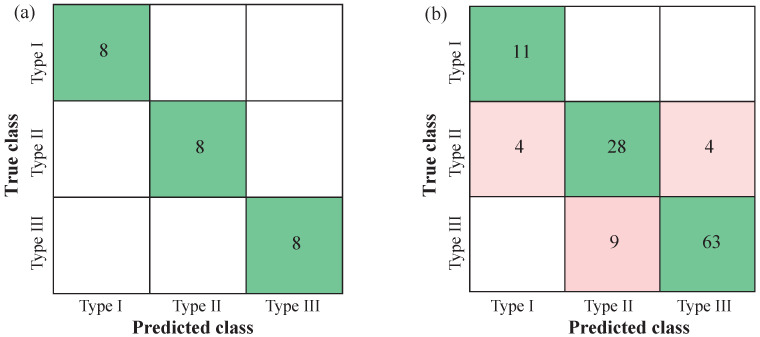
Confusion matrices for the most representative EGMs in Group 1 (**a**) and the whole database in Group 2 (**b**). All EGMs in Group 1 were correctly classified by their AF type, whereas 17 EGMs of Group 2 were wrongly classified.

**Figure 8:**
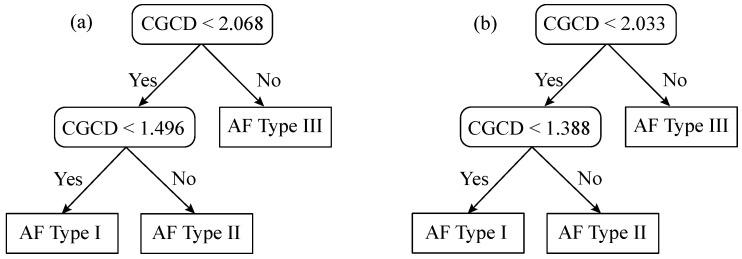
Decision tree together with thresholds obtained to classify EGMs by their AF Type through the application of CGCD. Scheme for the most representative EGMs in Group 1 (**a**) and for the whole database in Group 2 (**b**).

Classification tree analysis using 10-fold cross-validation showed 100% accuracy for Group 1 and 84.0–85.70% accuracy for Group 2, with 17 EGMs wrongly classified by their AF Type. These results are summarized on Table 2 and Figure 7. Mean CGCD values and standard deviation of AF Types of these two groups can be seen on Table 3, while the classification trees are shown on Figure 8. Note that wrongly classified EGMs of Group 2 were the most controversial regarding to their AF type classification. Still, in that case, CGCD was in consistency with their fractionation level.

**Figure 9:**
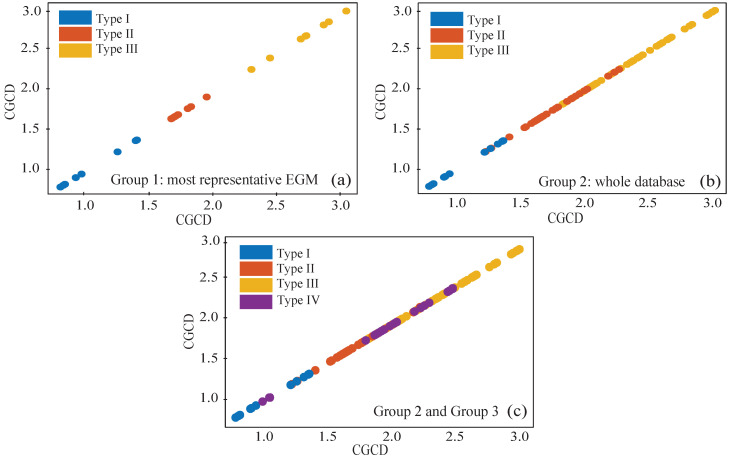
Scatterplots of CGCD values for the three AF Types in the most representative EGMs of Group 1 (**a**), in the whole database of Group 2 (**b**), and in Group 2 combined with the pseudo-real Type VI EGMs of Group 3 (**c**).

Finally, the scatterplots of Groups 1, 2, and combined Groups 2 and 3 can be seen on Figure 9. Values of the three AF Types are clearly distinguished in Group 1 by CGCD. Furthermore, although in Group 2 there are some misplaced AF Types II and III values, there is a clear pattern discriminating among the three AF Types as well. As expected, median CGCD values of AF Type IV in Figure 9c do not occupy a specific area, but span through the AF Types I–III values.

## 4. Discussion

This study showed that CGCD can estimate reliably the different fractionation levels that are present in AF EGMs. The idea of a metric that can faithfully quantify AF fractionation, and therefore organization of AF dynamics has been investigated in the past [59,60]. However, there is still need for a robust fractionation index that can operate optimally in short execution time and independently of parameters setting, so that it can be efficiently used for mapping the atrial substrate in AF.

One of the first works addressing a method to discriminate between different AF Types applied cluster and spectral analysis to 6 s duration EGMs and defined the best set of four parameters, presenting statistically significant differences between the main three AF Types [61]. The study recruited, nevertheless, a low proportion of AF Type III EGMs. As AF Type III is the most complex case of EGM fractionation and could indicate candidate ablation targets, this class should be emphasized in these kind of studies. Similarly, a newer study applied a set of various parameters, including time and spectral domain and morphological analysis in 4 s length signals [62]. Although it presented notably high classification accuracy with an optimal subset of seven parameters, the proportion of AF Type III EGMs was 35% of the overall dataset. As discrimination gets more complicated as the signal fractionation increases, a higher number of AF Type III EGMs would be necessary to verify the excellence of the classification accuracy presented in the aforementioned study.

In the same context, Kirchner et al. [63] applied principal component analysis (PCA) and cluster analysis for the discrimination between regular and irregular AF types of 90 s duration EGMs. With regular types being AF Type I EGMs and irregular types being AF Types II and III EGMs, the classification accuracy as high as 93% presented in this study misses the most significant and complicated part, the discrimination between AF Types II and III EGMs. Additionally, although these studies report results of high interest, no information on how the presented methods could be implemented on mapping devices is provided. With the shortest signal duration being 4 seconds, real-time mapping of AF using these methods is difficult to be accomplished.

Another work attempting to estimate the degree of AF fractionation involved atrial EGMs of 1.5 s duration that were recorded before the CA procedure [59]. The algorithm was based on the wavelet transform and the second differentiation of segments with continuous electrical activity, to find inflection points and compute the fractionation index (FI). Correlation between manual classification and the FI on highly fractionated EGMs yielded 81.8% sensitivity and 90.2% specificity, being these values poorer than the 87.5% sensitivity and 93.62% specificity that the present work achieved for the whole dataset of highly fractionated EGMs of Type III AF.

Haley et al. [60] developed an automated algorithm for the quantification of the percentage of AF fractionation in both paroxysmal and persistent AF EGMs of 4 s duration. The assessment of the algorithm was based on the correlation between the experts’ and the algorithm’s estimation of fractionation and achieved 77% sensitivity and 80% specificity. However, this study included paroxysmal AF patients (66.6% of the study population), which tend to have less complex AF EGMs and thus, automatic classification becomes significantly easier.

Nonlinear methods have been used by different studies for the assessment of AF organization, in terms of mean entropy [64] and correlation entropy [40], CorDim [43], and CGCD [40,44]. CorDim was found to discriminate between different levels of atrial organization in an AF-induced biophysical model [43]. CGCD in right atrial EGMs of persistent AF patients was analyzed before and after CA, and a relationship between the AF organization expressed as CGCD before the CA and the termination of AF after the CA was revealed [44].

The most interesting previous work to our study was performed by Hoekstra et al. [40], which applied CGCD analysis on right atrial unipolar EGMS of four seconds. They found that CGCD was able to distinguish among different AF Types. In spite of the fact that a promising application of CGCD to AF EGMs was revealed, this work contained only unipolar EGMs, which are quite unusual in daily electrophysiological procedures of AF ablation. In addition, the risk of significant ventricular contamination under unipolar recordings is high [65], and despite the QRS subtraction that was performed, four seconds is not an adequate interval for an effective ventricular removal [40,66]. As ventricular deflections can be even larger than atrial in unipolar EGMs, the corresponding CGCD values may very likely appear altered.

Bearing in mind all the aforementioned considerations, this is the first complete study to involve atrial bipolar EGMs and to perform analysis on 1 s segments with CGCD, establishing the optimal parameters that can be chosen for the highest performance. Being able to operate in small time segments is a very important parameter, as analysis intervals affect both the functionality and the performance of the algorithm, when it has to be applied in real-time. For this purpose, analysis on segments of 1.5 s and 0.5 s duration has also been applied. Classification accuracy was 76.5% using coarse-tree analysis and 10-fold cross-validation for the case of 1.5 s segmentation and 80.7% for the 0.5 s segmentation case. AF Type III was discriminated from the other two AF types using a one-vs-all ROC analysis with Sensitivity and Specificity of 86.11% and 89.30%, respectively, in the 1.5 s segmentation analysis, whereas Sensitivity and Specificity values for the equivalent 0.5 s case were 91.67% and 85.11%, respectively. Sensitivity and Specificity for the discrimination of AF Type I from the other two types were 96.3% and 100% respectively in both cases. As the aforementioned values did not overpass the analysis using 1 s window length, the latter segment was chosen for the analysis presented in this study. Moreover, in fractionation analysis, the stability of the recording catheter is an important issue [59] and analyzing in 1 s segments eliminates this barrier, facilitating the recording and shortening the whole procedure duration.

Selection of computational parameters is another issue that should be taken into consideration when the CGCD is estimated. So far, none of the previous works have agreed to the parameters being used, whereas time-series of different size were analyzed. Using 1 s fixed segments removes this ambiguity, as the parameters with the highest discriminative power can be chosen and incorporated as global computational parameters. To this respect, the short-time analysis introduced in the present work allows the method to catch sudden changes in AF fractionation. As it has been shown in Section 3, AF Type IV can be easily detected by second-to-second analysis. Furthermore, when the median CGCD value over segments longer than 1 s of AF Type IV EGMs is used, there is no discrimination of AF Type IV from the other three AF Types and it can be misclassified either as AF Type II or AF Type III. This means that in other methods involving larger analysis times, AF Type IV would be very likely ignored.

Apart from the aforesaid advantages of opting for 1 s analysis, this decision may involve the hazard of obtaining infinite CGCD values, in case that computational parameters are not carefully chosen. More detailed explanations about how CorDim and CGCD can be used in small data segments is included in the Appendix A. After multiple trials with different parameters, this work found the optimal computational parameters for the 1 s analysis to be m=4 for the embedded dimension, τ=8 ms for the time lag, and $N_{ref}=334$ for the reference points. Even with a relatively low embedded dimension, the algorithm could discern different fractionation levels, expressing a trustworthy comparison between them. It is necessary, however, to adjust the threshold values of different AF levels and to be able to understand what do these thresholds mean. This study found a threshold of 1.388 to discriminate between AF Type I and AF Type II and a threshold of 2.033 for the discrimination of AF Types II and III, for the case where the whole dataset is used. This does not mean that dynamics in AF Type III are 2-dimensional. Thresholds are only used in order to distinguish the degree of fractionation and are strongly linked to computational parameters, which in turn are a trade-off between optimal performance and functionality.

## 5. Limitations

The uneven choice of data size among the three AF Types may arise some oppositions to the robustness of this work. For this reason, it is necessary to explain the motivations of this choice. As mentioned in Section 2.1, AF type I is characterized by organized signals, with a clear, almost isoelectric line in between the atrial activations. Given the carefully chosen computational parameters, reconstruction of AF type I signals is not complex and the corresponding CGCD values are located in a well-defined region. In fact, AF Type I EGMs could be missed from the analysis without any change at all in the CGCD performance, since all EGMs of this category were correctly classified using 10-fold cross-validation. It was therefore decided to keep the proportion of AF Type I EGMs at almost 10% with respect to the whole dataset. Regarding the AF type II EGMs, as they almost span both AF Type I and AF Type III, involving a relatively low but significant percentage of 30% of the overall data size would add up the fidelity of the fractionation index, without distracting from the real challenge, which is to discriminate in high-fractionation environments.

Pre-selection of the most indicative EGMs (Group 1) as the first step of the analysis may be also criticized. Creation of this group allowed us to observe the performance in a completely controlled environment and ensure that the algorithm works in perfect consistency with the fractionation degree of the AF EGMs selected and annotated by expert physicians. In the next step, analysis included the whole dataset, which contains highly ambiguous EGMs and it was still operating optimally. In addition, thresholds for the AF Type IV EGMs detection were extracted from the whole dataset analysis (Group 2), as it is a more representative ensemble.

This work reported previous studies applying CGCD techniques in AF EGMs, providing a brief description of their aim. However, the dataset and CGCD methodology used in this work are different from these studies and therefore, a straightforward comparison between the presented study and these ones is not possible.

## 6. Conclusions

The present work has introduced, for the first time, the validity of CGCD as a reliable index to automatically estimate fractionation of bipolar AF EGMs. The use of epochs of 1 s length has facilitated the optimal setting of the CGCD computational parameters. The method has provided a higher classification ability than previous works dealing with the same challenging problem. Furthermore, calculation over 1 s segments provides short-time information and can agilely detect sudden changes in fractionation level of AF EGMs, leading to AF Type IV identification. Finally, given its short-time operation capabilities, this methodology could be used for mapping the atrial substrate, thus assisting ablation procedures under real-time settings for atrial substrate modification.

## Figures and Tables

**Table 1:** Statistical differences between the median CGCD values to discriminate between the three AF Types as well as for pairs of AF Types of Groups 1 and 2.

AF Types	Group 1	Group 2
AF Types I-II-III	*p* = 0.00004	*p* < 0.000010
AF Types I vs. II/III	*p* = 0.00010	*p* < 0.000010
AF Types III vs. I/II	*p* = 0.00010	*p* < 0.000010

**Table 2:** Classification accuracy by coarse decision tree for Groups 1 and 2 and the corresponding thresholds for the discrimination by different AF Types. $T_{1}$, $T_{2}$, and $T_{3}$ are the thresholds for discriminating AF Types I, II, and III, respectively.

Group	Nr of EGMs	Accuracy	Wrongly Classified	Threshold
1	24	100%	0	$T_{1}:<1.4958$ $T_{2}:\geq 1.4958,<2.0680$ $T_{3}:\geq 2.0677$
2	119	84.00–85.70%	17	$T_{1}:<1.3880$ $T_{2}:\geq 1.3880,<2.0326$ $T_{3}:\geq 2.0326$

**Table 3:** Mean and standard deviation of CGCD values of Groups 1 and 2 for AF Types I, II, and III. Results are presented as mean ± standard deviation.

AF Type	Group 1	Group 2
Type I	1.0220±0.2499	1.0608±0.2413
Type II	1.7097±0.0934	1.7587±0.2576
Type III	2.6406±0.2366	2.3884±0.3524

## Appendix A. Phase-Space Reconstruction and Correlation Dimension Using Small Data Size

Let $X=(x_{1},x_{2},\ldots ,x_{N})$ be a time-series of length *N*, as described in Section 2.2. Its signal reconstruction in the *m*-th phase-space will contain N−(m−1)·τ vectors. The *i*-th vector of the signal can be seen in Equation (1). In this study, *N* was equal to 1000 sample points, dimension *m* was equal to 4, and τ to 8 sample points. As sampling frequency was 1 kHz, each sample equals to 1 ms. Therefore, the first vector element in the signal will have coordinates **Y**${}_{1}^{4}=\;\left(x_{1},x_{9},x_{17},x_{25}\right)$ [67]. The reconstructed signal, will then look like the (ii) case of Figure 2.

As length of the reconstructed signal gets shorter in higher embedded dimensions and time lags, selection of these parameters should be done in consistency with the data size. This is a major problem for the most studies, as data of different sizes require different parameter values. The choice should be a trade off between preserving as many sample points and therefore as much information as possible and choosing a dimension into which dynamics of the attractor can be unfold.

The problem when using a small data size is that the range of embedded dimensions that can be used is limited and, as a result, the CorDim of the attractor can be significantly underestimated, as there is no sufficient space to span. A trustworthy calculation for the CorDim is that it lies well below $2\;\cdot \;log_{10}N$. As a result, for a data size of *N* = 1000 samples, a CorDim of 6 is the maximum that can be safely calculated [68]. It is, however, impossible to know a priori the dimension of the dynamics of the attractor, and consequently impossible to know if the length of the given time-series will be sufficient. Although a small embedded dimension may suppress the dynamics so that they look smaller than they really are, the signal reconstruction is still not free of the effect of the attractor [69]. This means that when the signal is reconstructed, even when using small parameters, the dynamics are preserved yet not fully expressed. This can be easily visualized from the resemblance of the reconstructed signal to the original one, even in the complicated AF Type III cases, as seen in Figure 2 case (ii) [67].

Of course this is a problem when we want to measure the CorDim, as there is a high risk of mistaken estimations. As described in Section 2.2, the estimation of this parameter is done by looking for linear regions in the plot of $ln(C^{\left(m\right)}\left(r\right))$ as a function of ln(r) for different values of *m*. The value of each one of that curves at a distance *r* results from the average of the $N_{ref*}$ points, which are the vectors from $N_{ref}$ (where $N_{ref*}\leq N_{ref}$) for which Θ=1 for that specific *r*. In other words, only a specific number ($N_{ref*}$) of the $N_{ref}$ vectors that are closer to each other than a distance *r* will participate in the plot. When the dimension of the dynamics is bigger than the embedded dimension, the number of points will scale for different values of *r*. When the embedded dimension is big enough and the dynamics are completely expressed, the number of points does not vary significantly and the curve becomes smoother. This is where the CorDim is computed at. As data size becomes smaller, $N_{ref}$ and $N_{ref*}$ become smaller as well. This leads to shorter scaling regions and the CorDim is wrongly computed to a smaller value [69].

This problem can be overcome with the use of CGCD, instead of CorDim. When CGCD is calculated, values obtained do not represent the real dimension of the underlying dynamics. Instead, it serves as a comparative measure between dynamics of different classes. This way, the dependence on the data size is completely avoided. On the contrary, the use of small datasets is even preferred in nonstationary dynamical systems, as they can follow the changing dynamics of the time-series [69]. It is therefore highly recommended the use of analysis segments as short as 1 s to catch short-life patterns and emancipate from the problem of varying computational parameters.
